# Outcomes of real-ear measurement-based fitting in unilateral hearing aid users: Influence of contralateral hearing

**DOI:** 10.1371/journal.pone.0358875

**Published:** 2026-09-24

**Authors:** Jeon Mi Lee, Tae Hoon Kong, Jeong-woo Lee, Hyun Jin Lee

**Affiliations:** 1 Department of Otorhinolaryngology, Ilsan Paik Hospital, Inje University College of Medicine, Goyang, Korea; 2 Department of Otorhinolaryngology-Head and Neck Surgery, Yonsei University Wonju College of Medicine, Wonju, South Korea; 3 Department of Medical Informatics and Statistics, Yonsei University Wonju College of Medicine, Wonju, South Korea; 4 Department of Otorhinolaryngology-Head and Neck Surgery, Incheon St. Mary’s Hospital, College of Medicine, The Catholic University of Korea, Seoul, Korea; LSU Health Shreveport, UNITED STATES OF AMERICA

## Abstract

**Objectives:**

Real-ear measurement (REM) is commonly used to verify hearing aid fittings; however, its added benefit over non-REM fitting in unilateral users and the influence of contralateral ear hearing remain unclear. In this retrospective study, we compared short-term audiometric and patient-reported outcomes between an REM-based fitting pathway and a manufacturer-first-fit pathway followed by routine clinical fine-tuning in unilateral hearing-aid users, and examined associations with contralateral-ear hearing.

**Methods:**

We retrospectively analyzed data from 105 unilateral hearing aid users with 3 months of follow-up (REM: n = 60; non-REM: n = 45). Baseline measures included pure-tone averages (PTA4) of the aided ear (i.e., the ear fitted with the hearing aid) and contralateral ear (i.e., the opposite ear), as well as the Hearing Handicap Inventory for the Elderly (HHIE). Outcomes included sound-field functional gain (ΔPTA4), achievement of ≥10 dB gain, change in HHIE (ΔHHIE; responder ≥18-point reduction), and the International Outcome Inventory for Hearing Aids (IOI-HA) scores at 3 months. Group comparisons were performed using non-parametric tests, and multivariable linear and logistic regression models were used to adjust for demographic and audiometric covariates. Associations between contralateral ear hearing and outcomes were examined using correlation and subgroup analyses.

**Results:**

Baseline characteristics were generally comparable between groups, although fitted side differed. At 3 months, mean functional gain was 11.0 ± 10.6 dB HL in the REM group and 9.1 ± 10.1 dB HL in the non-REM group (p = 0.2145). Mean ΔHHIE was 25.7 ± 30.3 and 32.9 ± 28.6 points (p = 0.1680), and mean IOI-HA scores were 24.4 ± 6.7 and 23.9 ± 5.9, respectively (p = 0.9492). In adjusted analyses, the REM-based fitting pathway was not associated with ΔHHIE (β = 1.97; 95% CI, −3.12 to 7.06) or 3-month IOI-HA score (β = 0.32; 95% CI, −2.23 to 2.86). Worse contralateral-ear PTA4 was associated with greater HHIE improvement (r = 0.389, p < 0.001).

**Conclusion:**

In this retrospective cohort of unilateral hearing-aid users, an REM-based fitting pathway was not associated with statistically significant 3-month audiometric or patient-reported outcome differences compared with manufacturer first-fit followed by routine fine-tuning; this does not establish equivalence or isolate the effect of REM. Contralateral-ear hearing was associated with perceived handicap improvement and may be relevant to outcome interpretation and counseling.

## Introduction

Hearing loss is one of the most prevalent chronic health conditions worldwide, significantly affecting communication, social participation, and quality of life [1,2]. More than 430 million people (over 5% of the global population) are estimated to have disabling hearing loss, and this number is projected to increase in the coming decades [1–3]. In older adults, hearing impairment is associated not only with communication difficulties but also with cognitive decline, social isolation, and depressive symptoms [4]. Hearing aids are the most widely used and effective intervention for sensorineural hearing loss; however, their benefits depend strongly on the quality of fitting [5,6]. Proper fitting optimizes speech audibility, comfort, and user satisfaction, whereas suboptimal fitting is associated with limited daily use, reduced perceived benefit, and abandonment of hearing aids [6]. Consequently, accurate, individualized fitting has become a key focus of evidence-based hearing rehabilitation.

Real-ear measurement (REM), also known as probe-microphone verification, is considered the gold standard method for confirming that hearing aid output matches prescriptive targets in the individual ear canal [7–9]. By directly measuring sound pressure near the tympanic membrane, REM provides a more precise representation of delivered amplification than manufacturer-derived simulations or coupler-based measures do [7,8]. Professional bodies, such as the American Speech-Language-Hearing Association and the British Academy of Audiology, recommend the routine use of REM in adult hearing aid fitting. Systematic reviews report small-to-moderate improvements in speech intelligibility, self-reported listening ability, and patient preference for REM-based fittings compared with initial-fit settings [8,10].

Despite these recommendations, REM remains underused in routine practice, and many fittings rely solely on manufacturer first-fit algorithms without objective verification [10,11]. This gap raises important questions regarding the real-world incremental value of REM, particularly in typical clinical populations where time and resource constraints are common. Furthermore, hearing aid outcomes depend strongly on individual factors, such as the degree and configuration of hearing loss, asymmetry, and the hearing status in the contralateral ear [5,11]. In unilateral fittings, the contralateral ear may play a critical role in determining subjective benefit, potentially overshadowing the contribution of optimization on the fitted side [11,12].

The benefits of hearing aids are commonly evaluated using a combination of objective and subjective measures. Objective outcomes include changes in pure-tone thresholds or aided sound-field performance. In contrast, subjective benefit is assessed using validated questionnaires, such as the Hearing Handicap Inventory for the Elderly (HHIE) and the International Outcome Inventory for Hearing Aids (IOI-HA) [13–16]. These instruments provide complementary information on perceived communication difficulties, hearing aid effectiveness, and changes in the quality of life.

Prior studies demonstrate acoustic and prescriptive advantages of REM; however, evidence directly linking REM-based fitting to short-term functional improvement and patient-reported outcomes in unilateral adult users remains limited, particularly in Asian clinical settings [11,16,17]. Additionally, the contribution of contralateral ear hearing to these outcomes has not been well characterized [14].

Therefore, in this retrospective study, we aimed to compare sound-field hearing thresholds, hearing-related handicap, and satisfaction between two routine clinical fitting pathways in individuals using a hearing aid in one ear: an REM-based fitting pathway and a manufacturer-first-fit pathway followed by routine clinical fine-tuning. Using the audiometric functional gain (ΔPTA4), HHIE, IOI-HA, and audiometric data, we compared outcomes between REM-verified and non-REM fittings and examined the role of contralateral ear hearing in shaping these outcomes. This approach was intended to describe whether these two fitting pathways were associated with different short-term outcomes in routine unilateral hearing-aid practice and to explore patient factors associated with real-world benefit.

## Materials and methods

### Study design and participants

This retrospective observational study was conducted at Incheon St. Mary’s Hospital, The Catholic University of Korea. We extracted clinical data from the electronic medical records of patients who underwent hearing aid fitting between January 1, 2022 and December 31, 2024. The data were accessed for research purposes between February 13, 2026 and March 31, 2026, and all data were fully anonymized prior to analysis. As all data were generated before protocol development, the study qualified as a retrospective chart review. The study protocol was approved by the Institutional Review Board of Incheon St. Mary’s Hospital (IRB No. OC25RASI0078), and the requirement for written informed consent was waived.

Patients were eligible if they were 19 years of age or older and received a unilateral hearing aid fitting during the study period. Only patients with unilaterally fitted ears were included; those fitted bilaterally or converted to bilateral fitting during follow-up were excluded from the study. For clarity, the ear fitted with the hearing aid is referred to as the aided ear, whereas the opposite ear is referred to as the contralateral ear.

Inclusion criteria were as follows: (1) at least one follow-up visit approximately 3 months after fitting, including routine hearing aid adjustments; (2) completion of pre-fitting and 3‑month post-fitting HHIE and/or IOI-HA questionnaires; (3) availability of conventional pure-tone audiometry before fitting and aided sound-field thresholds after fitting, with thresholds recorded at 0.5, 1, 2, and 4 kHz; and (4) unilateral hearing aid fitting in an ear with hearing thresholds that were poorer than or comparable to those of the contralateral ear. Patients were excluded if they had (1) a diagnosis of central auditory disorders (e.g., auditory neuropathy or other retrocochlear pathology), (2) missing or incomplete questionnaire data at baseline or follow-up, or (3) implantable hearing devices such as cochlear implants or bone-anchored hearing aids.

### Hearing aid fitting and REM procedure

Participants were classified into two groups based on whether REM was used during the fitting of the hearing aid: a REM and a non-REM group. Group allocation reflected a change in routine clinical practice following the introduction of the REM system and was therefore not randomized. The REM system was incorporated into the routine fitting protocol on March 2, 2023. All participants in the non-REM group (n = 45) were fitted before this date, whereas all participants in the REM group (n = 60) were fitted on or after this date. No non-REM fittings were performed after implementation of the REM system. Before implementing the REM system, hearing aids were fitted using the manufacturer’s fitting software with the National Acoustic Laboratories–Non-Linear 2 (NAL-NL2) prescription formula followed by routine clinical fine-tuning. After implementation, REM verification became part of the standard fitting protocol. For patients in the REM group, REM was performed using a clinical probe-microphone system (Trumpet, Inventis, Padova, Italy) in conjunction with Starkey’s REM Target Match workflow within the Inspire/Pro Fit fitting software. All fittings used the NAL-NL2 prescription formula as the default gain target. Hearing aids from Starkey, Widex, and Phonak were included. The manufacturer distribution was recorded separately for the REM and non-REM groups and is reported in Supplementary S1 Table. For all devices, NAL-NL2 was selected as the prescriptive rationale for the initial fitting. Throughout the study period, the same board-certified otolaryngologist personally performed the initial hearing-aid fitting, counseling, and subsequent fine-tuning for all 105 participants. All REM procedures in the REM group were performed by the same otolaryngologist.

For REM-based fittings, we inserted a probe tube into the ear canal and positioned it approximately 5 mm lateral to the tympanic membrane, in accordance with standard clinical guidelines. We measured real-ear aided responses in a sound-treated room using speech-shaped or speech-weighted stimuli presented at multiple input levels (typically 55, 65, and 75 dB sound pressure level [SPL]). Hearing aid gain was iteratively adjusted so that the real-ear insertion gain closely matched NAL-NL2 prescriptive targets at key frequencies across medium-level input (65 dB SPL) and did not show significant underfitting at soft or loud levels. Fitting was considered acceptable when the real-ear response at 65 dB SPL was within ±5 dB of the target at three or more of these frequencies. In the non-REM group, the initial settings generated by each manufacturer’s proprietary fitting software after selection of NAL-NL2 were used without probe-microphone verification. Thus, NAL-NL2 served as a common prescriptive framework, but manufacturer-specific fitting algorithms, default settings, and signal-processing configurations may have resulted in different initial device outputs. In both groups, follow-up visits were routinely scheduled immediately after fitting and at approximately 1 and 3 months after fitting. The same otolaryngologist performed fine-tuning at these visits using patient-reported concerns and listening preferences as the clinical basis for adjustment. The magnitude and frequency-specific content of gain adjustments, program changes, and final programmed settings at the 3-month assessment were not retained in a standardized analyzable format.

### Outcome measures

Pure-tone audiometry was performed in a sound-attenuated booth using calibrated clinical audiometers and supra-aural or insert earphones as appropriate. Air-conduction thresholds were obtained at standard test frequencies, and the four-frequency pure-tone average (PTA4) was defined as the average of thresholds at 0.5, 1, 2, and 4 kHz. For the aided ears, sound-field aided thresholds were measured in the aided condition using warble tones presented from a loudspeaker at 0° azimuth. The contralateral ear was masked during testing to minimize its contribution to the measured thresholds. Sound-field PTA4 was calculated using the same frequencies. Functional gain was quantified as the difference between unaided PTA4 and aided PTA4 (ΔPTA4 = unaided air-conduction PTA4–aided sound-field PTA4). Participants were classified into two groups based on the PTA4 of the contralateral ear: a good-ear group (contralateral ear PTA4 <40 dB) and a poor-ear group (contralateral ear PTA4 ≥40 dB).

Hearing handicap and hearing aid outcomes were assessed using the HHIE and IOI-HA. The HHIE is a validated 25-item questionnaire that assesses the perceived emotional and social consequences of hearing loss, with total scores ranging from 0 to 100; higher scores indicate greater hearing-related handicap. The IOI-HA is a 7-item questionnaire that evaluates overall hearing aid outcomes, with total scores ranging from 7 to 35; higher scores indicate greater perceived benefit and satisfaction. The HHIE was administered before hearing aid fitting and again at the 3‑month follow-up; change in HHIE (ΔHHIE) was calculated as baseline score minus the 3‑month score. A reduction ≥18 points on the HHIE was considered a clinically significant improvement, based on prior randomized trials and evidence reviews that reported a minimal significant difference of approximately 18–19 points for this instrument.

The IOI-HA was administered 3 months after fitting. The 3-month assessment was selected as a clinically pragmatic early follow-up time point and was consistent with the routine follow-up schedule in our clinical practice. The same follow-up interval was used for the HHIE and IOI-HA to ensure consistency across outcome measures. The IOI-HA total score at 3 months was used as the primary measure of hearing aid outcome and included as an adjustment variable in multivariable analyses.

### Statistical analysis

All statistical analyses were performed using IBM SPSS Statistics, version 21.0 (IBM Corp., Armonk, NY, USA). Continuous variables were summarized as means ± standard deviations; and categorical variables, as frequencies and percentages. For between-group comparisons (REM vs. non-REM), we used the Mann–Whitney U test for continuous variables and the chi-square test for categorical variables, as several outcomes were not normally distributed. To examine associations between fitting pathway and outcomes after adjustment for prespecified covariates, we applied multivariable linear regression for continuous outcomes (ΔHHIE and IOI-HA at 3 months) and multivariable logistic regression for binary outcomes (ΔPTA4 ≥ 10 dB and ΔHHIE ≥18 points). Covariates were selected *a priori* based on their established clinical relevance and on previous literature regarding hearing aid outcomes. All models were adjusted for age, sex, unaided PTA4 of the aided ear, contralateral ear PTA4, and fitted side. Baseline HHIE was additionally included in the ΔHHIE and HHIE responder models, whereas the 1-month IOI-HA score was additionally included in the 3-month IOI-HA model [11].

Pearson correlation analyses and subgroup comparisons were conducted to assess associations among contralateral ear hearing, frequency-specific threshold changes, and subjective outcomes. The frequency-specific analyses were considered exploratory and were performed to identify potential frequency-specific patterns, rather than to formally test prespecified hypotheses. To reduce the risk of false-positive findings, p-values from the seven frequency-specific adjusted comparisons were corrected using the Holm–Bonferroni procedure. A *p*-value <0.05 was considered statistically significant.

## Results

### Baseline demographic and audiological characteristics

A total of 105 unilateral hearing aid users were included (REM group: n = 60; non-REM group: n = 45). Demographic and audiologic characteristics were comparable between groups (Table 1). The mean age was 71.4 ± 10.7 years in the non-REM group and 69.9 ± 11.3 years in the REM group (*p =* 0.51). Unaided PTA4 of the aided ear was 55.1 ± 12.7 dB hearing level (HL) in the non-REM group and 55.7 ± 13.9 dB HL in the REM group (*p =* 0.85). The contralateral ear PTA4 was also similar between groups (42.3 ± 17.6 vs. 42.9 ± 15.4 dB HL, *p =* 0.94). Baseline HHIE scores tended to be higher in the non-REM group than in the REM group (59.3 ± 28.2 vs. 48.9 ± 28.5 points, *p =* 0.08); however, the difference was not statistically significant. The distributions of sex and contralateral ear hearing status did not differ significantly. However, the fitted side differed between groups, with more right-ear fittings in the non-REM group and more left-ear fittings in the REM group (*p =* 0.045) (Table 1).

**Table 1:** Baseline characteristics of unilateral hearing aid users by fitting method.

Variable	non-REM (n=45)	REM (n=60)	*p*-value
Age (y)	71.38±10.68	69.90±11.32	0.5086
Unaided PTA4 of the aided ear (dB HL)	55.06±12.73	55.65±13.87	0.8459
Unaided SRT of the aided ear (dB HL)	51.63±11.74	51.82±14.32	0.9655
Unaided SDS of the aided ear (%)	77.33±20.48	82.09±17.07	0.2007
Contralateral ear PTA4 (dB HL)	42.25±17.57	42.94±15.40	0.9432
Baseline HHIE (points)	59.25±28.16	48.87±28.53	0.0839
Sex (M/F)	19/26	31/29	0.4464
HA side (Lt/Rt)	18/27	37/23	0.0452*
Contralateral ear status (Good/Poor)	19/26	30/30	0.5532

*p < 0.05PTA4=pure-tone average at 0.5, 1, 2, and 4 kHz; SRT = speech recognition threshold; SDS = speech discrimination score; HHIE = Hearing Handicap Inventory for the Elderly; HA = hearing aid; Lt = left; Rt = right

### Comparison of outcomes between the REM and non-REM groups

At 3 months, both groups demonstrated meaningful functional gain (Table 2A). The mean functional gain (ΔPTA4) was 9.1 ± 10.1 dB HL in the non-REM group and 11.0 ± 10.6 dB HL in the REM group (*p =* 0.21). The proportion of patients achieving functional gain ≥10 dB was 57.8% in the non-REM group and 65.0% in the REM group (*p =* 0.58) (Table 2A).

**Table 2:** Audiometric and patient-reported outcomes at 3 months by fitting method.

Outcome	non-REM (n=45)	REM (n=60)	*p-*value
**(A) Audiometric outcomes**			
ΔPTA4 (dB HL)	9.14±10.10	11.02±10.58	0.2145
Functional gain ≥10 dB, n (%)	26 (57.8)	39 (65.0)	0.5816
**(B) Patient-reported outcomes**			
ΔHHIE (points)	32.89±28.60	25.73±30.25	0.1680
HHIE responder ≥18 points, n (%)	28 (62.2)	31 (51.7)	0.3788
IOI-HA score at 3 months	23.91±5.87	24.43±6.72	0.9492

PTA4 = pure-tone average at 0.5, 1, 2, and 4 kHz; SF = sound field; HHIE = Hearing Handicap Inventory for the Elderly; IOI-HA = International Outcome Inventory for Hearing Aids.

Patient-reported outcomes were also similar between groups (Table 2B). HHIE scores improved by 32.9 ± 28.6 points in the non-REM group and 25.7 ± 30.3 points in the REM group (*p =* 0.17). The proportion of HHIE responders (≥18‑point reduction) was 62.2% in the non-REM group and 51.7% in the REM group (*p =* 0.38). IOI-HA scores at 3 months were comparable between groups (23.9 ± 5.9 vs. 24.4 ± 6.7, *p =* 0.95) (Table 2B). Overall, we observed no statistically significant between-group differences in either audiometric or patient-reported primary outcomes.

### Adjusted associations between fitting pathway and outcomes

To examine adjusted associations between fitting pathway and outcomes, we performed multivariable regression analyses adjusting for age, sex, unaided PTA4 of the aided ear, contralateral ear PTA4, fitted side, and outcome-specific baseline covariates (Table 3). In linear models, REM use was not significantly associated with ΔHHIE (*β* = 1.97; 95% confidence interval [*CI*]: −3.12–7.06, *p =* 0.445) or IOI-HA score at 3 months (*β* = 0.32; 95% *CI*: −2.23–2.86, *p =* 0.805). In logistic models, REM did not significantly predict functional gain ≥10 dB (odds ratio [*OR*]=1.48; 95% *CI*: 0.54–4.08, *p =* 0.448) or HHIE improvement ≥18 points (*OR*=1.19; 95% *CI*: 0.26–5.49, *p =* 0.823). These analyses did not identify statistically significant adjusted associations between the REM-based fitting pathway and the evaluated short-term outcomes (Table 3). However, the confidence intervals around these estimates were compatible with small differences in either direction.

**Table 3:** Multivariable regression analyses for the effect of REM-based fitting.

Dependent variable	*β* for REM (vs non-REM)	SE	95% *CI*	*p*-value
**(A) Linear regression (continuous outcomes)**				
ΔHHIE (points)	1.97	2.56	−3.12–7.06	0.445
IOI-HA at 3 months	0.32	1.28	−2.23–2.86	0.805
**Dependent variable**	***OR* for REM (vs non-REM)**		**95% *CI***	***p*-value**
**(B) Logistic regression (binary outcomes)**				
Functional gain ≥10 dB	1.48		0.54–4.08	0.4477
HHIE improvement ≥18 points	1.19		0.26–5.49	0.8234

All models were adjusted for age, sex, unaided PTA4 of the aided ear, contralateral ear PTA4, and fitted side. The ΔHHIE and HHIE improvement (≥18-point) models were additionally adjusted for baseline HHIE. The model for IOI-HA at 3 months was additionally adjusted for the 1-month IOI-HA score, used as the earliest available reference point since IOI-HA cannot be measured prior to hearing aid fitting. For functional gain (≥10 dB), no additional baseline term was required because unaided PTA4 of the aided ear, already included as a core covariate, served as the baseline reference for this outcome. REM = real-ear measurement; PTA4 = pure-tone average at 0.5, 1, 2, and 4 kHz; SE = standard error; *CI* = confidence interval; *OR*=odds ratio; SF = sound field; HHIE = Hearing Handicap Inventory for the Elderly; IOI-HA = International Outcome Inventory for Hearing Aids.

### Impact of contralateral hearing on subjective outcomes

Hearing in the contralateral ear showed a significant association with improvement in perceived handicap (Table 4). Worse contralateral ear PTA4 moderately correlated with greater HHIE improvement (*r =* 0.389, *p <* 0.001), whereas its correlations with HHIE at 3 months (*r =* 0.105, *p =* 0.29) and IOI-HA scores at 3 months (*r=*−0.053, *p =* 0.60) were not significant. When stratified by contralateral ear hearing, the poor-ear group exhibited significantly greater HHIE improvement than the good-ear group did (38.5 ± 30.1 vs. 17.4 ± 24.9 points, *p =* 0.004), whereas IOI-HA scores did not differ between groups (23.9 ± 5.5 vs. 24.6 ± 7.3, *p =* 0.55) (Table 4). Within each contralateral ear subgroup, no significant differences were observed between REM and non-REM fitting in ΔPTA4, ΔHHIE, or IOI-HA scores (all *p* > 0.15). These findings indicate that contralateral-ear PTA4 was associated with HHIE improvement in this cohort, whereas no significant between-group differences in the evaluated outcomes were observed according to fitting pathway.

**Table 4:** Impact of contralateral ear hearing on subjective outcomes.

Variable pair		*r*	*p*-value
**(A) Correlation between contralateral ear hearing and outcomes**			
Contralateral ear PTA4 vs ΔHHIE		0.389	0.000*
Contralateral ear PTA4 vs HHIE at 3 months		0.105	0.291
Contralateral ear PTA4 vs IOI-HA at 3 months		−0.053	0.598
**Outcome**	**Good-ear group (<40 dB, n=49)**	**Poor-ear group (≥40 dB, n=56)**	***p*-value**
**(B) Outcomes by contralateral ear hearing group**			
ΔHHIE (points)	17.40±24.94	38.50±30.07	0.004*
IOI-HA scores at 3 months	24.58±7.30	23.89±5.45	0.553

*p<0.05. PTA4=pure-tone average at 0.5, 1, 2, and 4 kHz; HHIE = Hearing Handicap Inventory for the Elderly; IOI-HA = International Outcome Inventory for Hearing Aids

### Frequency-specific improvements and associations with outcomes

In exploratory frequency-specific adjusted analyses, nominal associations were observed at 250 Hz and 8 kHz before correction for multiple comparisons (Table 5). The adjusted-model p-values at 250 Hz and 8 kHz were 0.0327 and 0.0385, respectively; however, neither association remained statistically significant after Holm–Bonferroni correction across the seven frequency-specific comparisons (both Holm-adjusted p = 0.2289). No other frequency-specific adjusted association was statistically significant. Correlations between frequency-specific changes and ΔHHIE were weak and non-significant across all frequencies. The correlation between 8-kHz improvement and the 3-month IOI-HA score had a nominal p-value of 0.035 but was exploratory and is presented descriptively.

**Table 5:** Frequency-specific improvements by fitting method and associations with outcomes.

Frequency	non-REM Δ (dB HL)	REM Δ (dB HL)	*p* (raw)	*β* for REM (adjusted)	*p* (adjusted)	Holm-adjusted *p*
**(A) Frequency-specific improvements by fitting method**						
250 Hz	5.00±13.98	9.50±15.20	0.1345	4.28	0.0327	0.2289
500 Hz	8.33±14.22	11.33±15.91	0.2368	2.75	0.1781	0.8905
1 kHz	15.22±10.55	15.00±14.05	0.9402	−1.34	0.4793	1.0000
2 kHz	15.00±10.28	13.83±12.26	0.6349	−1.14	0.5757	1.0000
3 kHz	11.22±11.88	9.83±12.75	0.7141	−1.08	0.6243	1.0000
4 kHz	8.00±12.27	8.25±12.00	0.6751	0.57	0.7990	1.0000
8 kHz	12.78±13.25	16.57±14.71	0.1974	5.09	0.0385	0.2289
**Variable pair**			***r***		***p*-value**	
**(B) Correlations between frequency-specific improvements and outcomes**						
ΔHHIE vs. Δ250 Hz			−0.025		0.7992	
ΔHHIE vs. Δ500 Hz			0.114		0.2510	
ΔHHIE vs. Δ1 kHz			0.134		0.1757	
ΔHHIE vs. Δ2 kHz			0.144		0.1448	
ΔHHIE vs. Δ3 kHz			0.186		0.0588	
ΔHHIE vs. Δ4 kHz			0.178		0.0702	
ΔHHIE vs. Δ8 kHz			0.140		0.1751	
IOI-HA 3M vs. Δ250 Hz			0.040		0.6868	
IOI-HA 3M vs. Δ500 Hz			0.083		0.4065	
IOI-HA 3M vs. Δ1 kHz			0.102		0.3054	
IOI-HA 3M vs. Δ2 kHz			0.148		0.1356	
IOI-HA 3M vs. Δ3 kHz			−0.062		0.5345	
IOI-HA 3M vs. Δ4 kHz			0.032		0.7512	
IOI-HA 3M vs. Δ8 kHz			0.218		0.0350	

Models were adjusted for age, sex, contralateral-ear PTA4, and baseline threshold at each frequency. Holm–Bonferroni correction was applied across the seven frequency-specific adjusted comparisons.PTA4 = pure-tone average at 0.5, 1, 2, and 4 kHz; HHIE = Hearing Handicap Inventory for the Elderly; IOI-HA = International Outcome Inventory for Hearing Aids; 3M = 3‑month follow-up.

## Discussion

In this retrospective cohort, the REM-based fitting pathway and the manufacturer-first-fit pathway followed by routine clinical fine-tuning showed no statistically significant differences in overall sound-field thresholds, self-reported hearing handicap, or hearing-aid outcome scores over 3 months. ΔPTA4, the proportion of patients achieving ≥10 dB functional gain, HHIE change, HHIE responder rates, and IOI-HA scores were comparable between the REM and non-REM groups in unadjusted and multivariable analyses. The observed effect estimates were small, although their confidence intervals did not exclude clinically relevant differences. Given the modest effect sizes reported in prior meta-analyses, our sample size may have limited statistical power to detect small but clinically significant differences between groups, raising the possibility of a type II error. Accordingly, the absence of statistically significant between-group differences should not be interpreted as evidence of equivalence between the fitting pathways or as evidence that REM verification has no independent effect.

Our results appear to be more conservative than those of Almufarrij et al., who reported small-to-moderate positive effects of REM on speech intelligibility, self-reported listening ability, and user preference in a systematic review and meta-analysis (standardized mean differences: 0.15–0.59 in favor of REM). However, that review also emphasized that the available evidence was based on relatively few studies with small samples and heterogeneous outcome measures, and whether the observed differences exceed minimal clinically significant differences for most instruments remains unclear [18]. In this context, the absence of a statistically significant REM effect on the HHIE or IOI-HA in our cohort may reflect a limited true effect size, measurement variability, and the influence of other patient-level factors. This interpretation is limited by the precision of the available estimates and by the retrospective pathway-based design. Because both groups underwent routine post-fitting fine-tuning based on patient feedback, the present study compares two clinical fitting pathways and cannot isolate the independent effect of REM verification. Although NAL-NL2 was selected as the common prescriptive rationale, manufacturer-specific first-fit implementations and default signal-processing settings may have produced different initial outputs across devices; therefore, their potential contribution to the observed outcomes cannot be excluded. Under these real-world conditions, comparable short-term outcomes may be achieved through appropriate follow-up adjustments to hearing aids, regardless of whether REM verification was initially performed.

In exploratory frequency-specific adjusted analyses, nominal associations were observed at 250 Hz and 8 kHz before correction for multiple comparisons. However, the unadjusted between-group comparisons at these frequencies were not statistically significant, and neither adjusted association remained statistically significant after Holm–Bonferroni correction across the seven frequency-specific comparisons. These observations should therefore be considered hypothesis-generating only and do not provide confirmatory evidence of frequency-specific benefit from the REM-based fitting pathway. Their interpretation is further limited by the absence of statistically significant differences in the overall audiometric and patient-reported outcomes

We observed an association between contralateral-ear hearing and change in perceived hearing handicap. Worse hearing in the contralateral ear was moderately associated with greater improvement in HHIE. Consistent with this finding, the poor-ear group, defined as participants with contralateral ear PTA4 ≥ 40 dB, demonstrated more than twice the HHIE improvement observed in the good-ear group, those with contralateral ear PTA4 < 40 dB. In contrast, IOI-HA scores did not differ significantly by contralateral ear subgroups. These findings suggest that overall hearing configuration may be associated with perceived benefit in unilateral fittings. One possible explanation is that patients with poorer contralateral-ear hearing may rely more on the amplified ear for daily communication; however, this potential mechanism could not be evaluated in the present retrospective study. Conversely, relatively better contralateral-ear hearing may be associated with less change in patient-reported handicap after unilateral fitting; however, potential mechanisms, including greater reliance on the contralateral ear or a ceiling effect, could not be determined from the present data. These observations align with recent large-scale data showing that hearing-aid fitting laterality and contralateral ear status are closely related to persistence and self-reported benefit in real-world users [11,19].

Collectively, these findings suggest that contralateral-ear hearing status was associated with short-term change in perceived handicap in this cohort; this association does not establish a causal or primary role relative to REM use. This interpretation does not diminish the technical importance of REM; rather, it underscores that its incremental contribution should be considered within the broader context of hearing asymmetry and patient reliance on the fitted ear. Clinically, this finding highlights the importance of individualized counseling. In clinical counseling, contralateral-ear hearing status may be considered when discussing the range of patient-reported benefit that may be observed after unilateral amplification. In contrast, patients with poorer hearing in the contralateral ear may experience greater subjective benefit from unilateral amplification but may also require closer monitoring to prevent potential auditory deprivation of the unaided ear over time [11,20,21]. In addition, cognitive function may have influenced adaptation to hearing aids and responses to patient-reported outcome measures in this predominantly older adult population. As cognitive status was not systematically assessed in the present study, its potential influence on subjective outcomes should be considered when interpreting our findings [4].

Certain limitations should be acknowledged. First, the retrospective design and single-center setting may limit generalizability and introduce selection bias. This was a retrospective study based on available clinical data; thus, no *a priori* sample size calculation was performed. Therefore, the study may have been underpowered to detect small but clinically meaningful differences between groups. The decision to perform REM was not randomized because group allocation was determined by the calendar period of hearing-aid fitting, before or after implementation of REM into routine practice on March 2, 2023. Therefore, in addition to patient-level selection bias, temporal or secular confounding cannot be excluded in this historically controlled comparison. Although the manufacturer distribution was broadly similar between groups, detailed model-level comparisons were not feasible because individual models were represented by small numbers of participants; therefore, residual confounding related to model-specific device characteristics or manufacturer-specific processing cannot be completely excluded. Second, we did not retain detailed real-ear target deviation data, which precluded analysis of the relationship between verification accuracy and outcome magnitude. In addition, detailed records of follow-up gain adjustments and final programmed settings at 3 months were not available in a standardized analyzable format; therefore, the relative contributions of initial fitting and subsequent fine-tuning could not be separated. Third, follow-up was limited to 3 months; therefore, we could not assess longer-term effects of REM on device-use persistence, real-world communication, or auditory deprivation. Fourth, although we included multiple covariates in the multivariable models, residual confounding from unmeasured factors—such as cognitive status, counseling quality, listening environments, and device-handling ability—remains possible. The frequency-specific analyses were exploratory and should be interpreted as hypothesis-generating. Although Holm–Bonferroni correction was applied across the seven frequency-specific adjusted comparisons, the study was not designed to test prespecified frequency-specific hypotheses; these findings require confirmation in adequately powered prospective studies.

Despite these limitations, this study provides real-world information on short-term outcomes associated with two routine fitting pathways in unilateral adult hearing-aid users. REM-based fitting was not associated with statistically significant between-group differences in global handicap or satisfaction scores, and contralateral-ear hearing was associated with change in perceived handicap. These findings suggest that outcome expectations for unilateral hearing aid users should incorporate hearing asymmetry and hearing status in the contralateral ear, and that REM should be viewed as a fundamental verification tool whose incremental impact depends on patient-specific auditory configuration. Future prospective studies with larger samples, longer follow-up, detailed REM accuracy metrics, and speech-in-noise assessments are needed to clarify which subgroups derive the greatest functional benefit from REM and how its clinical value can be maximized.

## Supporting information

S1 Table Distribution of hearing aid manufacturers in the REM and non-REM groups. (DOCX)

S1 Dataset De-identified individual participant dataset used for the statistical analyses reported in this study. (XLSX)

## References

[1] Olusanya BO, Neumann KJ, Saunders JE. The global burden of disabling hearing impairment: A call to action. Bull World Health Organ. 2014;92(5):367–73. doi: 10.2471/BLT.13.128728 24839326 PMC4007124

[2] Huang G-J, Fan Z-J, Lu B-Q. The global prevalence of complete hearing loss in 204 countries and territories from 1992 to 2021: A systematic analysis for the global burden of disease study 2021. Front Public Health. 2025;13:1526719. doi: 10.3389/fpubh.2025.1526719 40302771 PMC12039815

[3] Stephenson J. WHO Report Predicts Hearing Loss for 1 in 4 People Worldwide by 2050. JAMA Health Forum. 2021;2(3):e210357. doi: 10.1001/jamahealthforum.2021.0357 36218447

[4] Wu F, Zhou C. Hearing impairment and cognitive function: Mediating role of social isolation and depression. Am J Alzheimers Dis Other Demen. 2024;39:15333175241227318. doi: 10.1177/15333175241227318 38198589 PMC10785707

[5] Wu Y-H, Ho H-C, Hsiao S-H, Brummet RB, Chipara O. Predicting three-month and 12-month post-fitting real-world hearing-aid outcome using pre-fitting acceptable noise level (ANL). Int J Audiol. 2016;55(5):285–94. doi: 10.3109/14992027.2015.1120892 26878163 PMC4823154

[6] Houmøller SS, Wolff A, Tsai L-T, Narayanan SK, Hougaard DD, Gaihede ML, et al. Impact of hearing aid technology level at first-fit on self-reported outcomes in patients with presbycusis: A randomized controlled trial. Front Aging. 2023;4:1158272. doi: 10.3389/fragi.2023.1158272 37342862 PMC10277865

[7] Humes LE, Pavlovic C, Bray V, Barr M. Real-ear measurement of hearing threshold and loudness. Trends Amplif. 1996;1(4):121–35. doi: 10.1177/108471389600100402 25425857 PMC4172257

[8] Davidson A, Marrone N, Wong B, Musiek F. Predicting hearing aid satisfaction in adults: A systematic review of speech-in-noise tests and other behavioral measures. Ear Hear. 2021;42(6):1485–98. doi: 10.1097/AUD.0000000000001051 33883425

[9] Lee JM, Shin S-H, Suh M-W, Choi SJ, Shin Y-R, An Y-H, et al. A position statement on the treatment of hearing impairment, focused on hearing aids, from the Korean Otological Society Hearing Aid Study Group. J Korean Med Sci. 2025;40(23):e182. doi: 10.3346/jkms.2025.40.e182 40524627 PMC12170300

[10] Almufarrij I, Munro KJ, Dillon H. Does probe-tube verification of real-ear hearing aid amplification characteristics improve outcomes in adult hearing aid users? A protocol for a systematic review. BMJ Open. 2020;10(7):e038113. doi: 10.1136/bmjopen-2020-038113 32690533 PMC7371126

[11] Zobay O, Naylor G, Saunders GH, Dillard LK. Fitting a hearing aid on the better ear, worse ear, or both: Associations of hearing-aid fitting laterality with outcomes in a large sample of US veterans. Trends Hear. 2023;27. doi: 10.1177/23312165231195987 37615317 PMC10467180

[12] Arlinger S. Negative consequences of uncorrected hearing loss--A review. Int J Audiol. 2003;42 Suppl 2:2S17-20. doi: 10.3109/14992020309074639 12918624

[13] Newman CW, Weinstein BE. The Hearing Handicap Inventory for the Elderly as a measure of hearing aid benefit. Ear Hear. 1988;9(2):81–5. doi: 10.1097/00003446-198804000-00006 3366309

[14] Cox RM, Alexander GC. The International Outcome Inventory for Hearing Aids (IOI-HA): Psychometric properties of the English version. Int J Audiol. 2002;41(1):30–5. doi: 10.3109/14992020209101309 12467367

[15] Frisby C, Eikelboom RH, Mahomed-Asmail F, de Kock T, Manchaiah V, Swanepoel DW. International Outcome Inventory for Hearing Aids (IOI-HA) translation into isiXhosa. Int J Audiol. 2024;63(3):229–32. doi: 10.1080/14992027.2022.2154281 36519291 PMC11384185

[16] Humes LE, Dhar S, Singh J. Some considerations in the use of the Hearing Handicap Inventory for the Elderly and its derivatives as hearing-aid outcome measures. Int J Audiol. 2025;:1–20. doi: 10.1080/14992027.2025.2511218 40444943

[17] Almufarrij I, Dillon H, Adams B, Greval A, Munro KJ. Listening preferences of new adult hearing aid users: A registered, double-blind, randomized, mixed-methods clinical trial of initial versus real-ear fit. Trends Hear. 2023;27:23312165231189596. doi: 10.1177/23312165231189596 37942535 PMC10637150

[18] Almufarrij I, Dillon H, Munro KJ. Does probe-tube verification of real-ear hearing aid amplification characteristics improve outcomes in adults? A systematic review and meta-analysis. Trends Hear. 2021;25:2331216521999563. doi: 10.1177/2331216521999563 33899603 PMC8083001

[19] Noh H, Lee D-H. Predictable factors of people with asymmetrical hearing loss wearing a hearing aid in the worse ear only. J Clin Med. 2023;12(6):2251. doi: 10.3390/jcm12062251 36983249 PMC10059651

[20] Wieselberg MB, Iório MCM. Hearing aid fitting and unilateral auditory deprivation: Behavioral and electrophysiologic assessment. Braz J Otorhinolaryngol. 2012;78(6):69–76. doi: 10.5935/1808-8694.20120036 23306571 PMC9446341

[21] Naylor G, Dillard LK, Zobay O, Saunders GH. Associations between pre-fitting factors and 2-year hearing aid use persistence, derived from health records and post-fitting battery order data of 284,175 US veterans. Ear Hear. 2025;46(6):1595–602. doi: 10.1097/AUD.0000000000001694 40518564 PMC12533770

