# Supplementary material for: Outcomes of real-ear measurement-based fitting in unilateral hearing aid users: Influence of contralateral hearing
Source: PLoS One. 2026 Sep 24;21(9):e0358875. doi: 10.1371/journal.pone.0358875 (PMC13606958; doi:10.1371/journal.pone.0358875)
Supplement: Supplementary file 1 [file pone.0358875.s001.docx]

| **Variable** | **Non-REM (n=45)** | **REM (n=60)** | **p-value** |
| --- | --- | --- | --- |
| Manufacturer |  |  | 0.297 |
| Starkey | 22 (48.9) | 26 (43.3) |  |
| Widex | 23 (51.1) | 31 (51.7) |  |
| Phonak | 0 (0.0) | 3 (5.0) |  |
| Device type |  |  |  |
| RIC | 32 (71.1) | 55 (91.7) |  |
| CIC | 10 (22.2) | 5 (8.3) |  |
| BTE | 2 (4.4) | 0 (0.0) |  |
| ITC | 1 (2.2) | 0 (0.0) |  |

**Supplementary Table S1. Distribution of hearing-aid manufacturers and device types according to fitting pathway**

*Values are presented as n (%). Manufacturer distribution was compared using Pearson’s chi-square test. Because of sparse cells, this comparison should be interpreted cautiously. Device-type categories were descriptively reported and were not formally compared.*
